# The Global Immune–Nutrition–Inflammation Index Is Associated with Survival Outcomes and Enhances Prognostic Discrimination in Metastatic Pancreatic Cancer

**DOI:** 10.3390/medicina62071279

**Published:** 2026-07-02

**Authors:** Kamuran Yüceer, Oktay Bozkurt, Mevlüde Inanç, Metin Ozkan

**Affiliations:** Department of Medical Oncology, Erciyes University Faculty of Medicine, Kayseri 38039, Turkey; oktaybozkurt@erciyes.edu.tr (O.B.); mevludeinanc@hotmail.com (M.I.); metino@erciyes.edu.tr (M.O.)

**Keywords:** metastatic pancreatic cancer, systemic inflammation, global immune–nutrition–inflammation index, composite biomarkers, prognostic stratification, survival outcomes

## Abstract

*Background and Objectives*: Metastatic pancreatic ductal adenocarcinoma (PDAC) continues to carry a poor prognosis despite advances in treatment, underscoring the need for simple and accessible biomarkers that reflect tumor–host interactions. The Global Immune–Nutrition–Inflammation Index (GINI), which combines inflammatory, immune, and nutritional parameters, may offer improved prognostic stratification compared with conventional indices. *Materials and Methods*: This retrospective cohort study included 126 patients with metastatic PDAC treated between 2015 and 2024. GINI, neutrophil-to-lymphocyte ratio (NLR), systemic immune-inflammation index (SII), and systemic inflammation response index (SIRI) were calculated using baseline laboratory data. Discriminative ability was evaluated by receiver operating characteristic (ROC) analysis. Survival outcomes were assessed using Kaplan–Meier curves and Cox proportional hazards models. *Results*: Among the evaluated indices, GINI showed the best discriminative performance (AUC, 0.769; 95% CI, 0.637–0.900), with a sensitivity of 78.8% and specificity of 76.9%. Patients with lower GINI values had significantly longer overall survival than those with higher values (median OS, 11.0 vs. 7.0 months; *p* = 0.014). Although progression-free survival differed statistically (*p* = 0.006), median PFS was the same in both groups (5.0 months). In univariable analysis, higher GINI was associated with worse OS (HR, 1.67; *p* = 0.022) and PFS (HR, 1.75; *p* = 0.012). However, in multivariable analysis, ECOG performance status remained the only consistent independent predictor, and GINI was no longer significant. *Conclusions*: GINI is a practical and biologically meaningful biomarker that improves risk discrimination in metastatic PDAC. While it does not retain independent prognostic significance, its ability to capture the overall tumor–host interaction supports its use as a complementary tool for baseline risk assessment.

## 1. Introduction

Metastatic pancreatic ductal adenocarcinoma (PDAC) is one of the most lethal malignancies in oncology, characterized by rapid systemic dissemination, profound therapeutic resistance, and persistently poor survival outcomes [1]. Despite incremental advances in cytotoxic regimens, such as FOLFIRINOX and gemcitabine-based combinations, durable disease control remains rare [2]. Recently, emerging therapeutic strategies, including immune checkpoint inhibitors in biomarker-selected populations and novel targeted approaches such as pan-RAS inhibitors and antibody–drug conjugates, have generated renewed optimism. Early phase data, including those evaluating agents such as daraxonrasib, have demonstrated encouraging response rates, even in heavily pretreated settings [3]. Nevertheless, these advances have yet to meaningfully alter the overall trajectory of metastatic PDAC at the population level, where median survival remains limited, and inter-patient variability in outcomes remains substantial. This persistent gap underscores the need for clinically accessible biomarkers capable of capturing the biological heterogeneity underlying differential treatment responses.

Cancer progression is now widely viewed as the result of ongoing interactions between tumor-specific characteristics and the host’s systemic environment. In pancreatic ductal adenocarcinoma (PDAC), this relationship is especially evident, driven by metabolic alterations within tumor cells, a dense and immunosuppressive stromal background, and a systemic inflammatory state that is often accompanied by cachexia [4,5]. In this setting, composite biomarkers based on routine hematologic parameters have attracted interest as practical indicators of the tumor–host interaction. Indices such as the neutrophil-to-lymphocyte ratio (NLR), systemic immune-inflammation index (SII), and systemic inflammation response index (SIRI) have been repeatedly linked to survival outcomes across a range of cancers, including pancreatic cancer [6,7,8,9,10]. Despite this, their clinical value remains limited. These indices mainly capture specific aspects of inflammation rather than the full complexity of the host response. In addition, their shared biological components and the lack of standardized cutoff values lead to variability in results between studies, which reduces their consistency and limits their use in routine clinical practice.

The Global Immune–Nutrition–Inflammation Index (GINI) has recently emerged as a more comprehensive biomarker, combining measures of systemic inflammation, immune competence, and nutritional status into a single composite parameter [11]. By combining neutrophils, platelets, monocytes, and C-reactive protein with lymphocyte counts and albumin levels, GINI captures both pro-tumor inflammatory signaling and the host physiological reserve. Beyond oncology, the GINI has also been explored in cardiovascular and metabolic contexts, where it has been associated with systemic risk profiles and adverse outcomes. In cancer populations, emerging data across tumor types, including thoracic, gastrointestinal, and neuro-oncologic malignancies, suggest that GINI may provide improved prognostic discrimination compared with conventional indices [12,13,14,15,16]. However, its role in metastatic PDAC, a disease in which tumor–host interactions are particularly critical, remains unclear.

In this study, we aimed to evaluate the prognostic relevance of GINI in patients with metastatic pancreatic cancer and systematically compare its performance with that of established inflammatory biomarkers. We hypothesized that a composite index reflecting the integrated tumor–host interface would enhance risk discrimination beyond conventional inflammation-based metrics, thereby providing a more clinically informative framework for prognostic stratification in this highly aggressive disease.

## 2. Materials and Methods

### 2.1. Study Design and Patient Population

This retrospective cohort study included consecutive patients with histopathologically confirmed metastatic PDAC treated between 1 January 2015, and 31 December 2024, at the Erciyes University Faculty of Medicine. This study was designed to evaluate the prognostic relevance of systemic inflammatory and composite biomarkers in a contemporary metastatic treatment setting.

**Figure 1:**
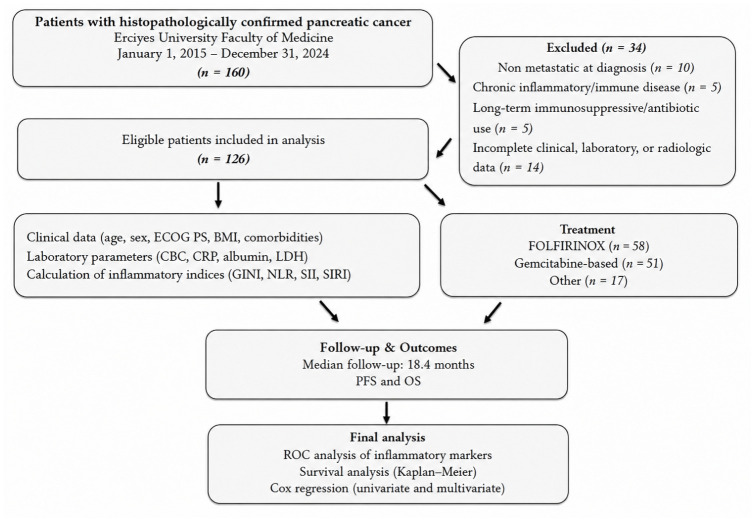
Flow diagram of patient selection, treatment allocation, and analysis in patients with metastatic pancreatic cancer.

A total of 160 patients were screened for eligibility. Patients were excluded if they had non-metastatic disease at diagnosis (*n* = 10), coexisting chronic inflammatory or autoimmune conditions, or exposure to systemic immunosuppressive therapies that could confound baseline inflammatory parameters (*n* = 10), or incomplete clinical, laboratory, or follow-up data (*n* = 14). To further enhance biological interpretability, cases with evidence of acute inflammatory perturbations at the baseline were excluded. The final analytic cohort comprised 126 patients (Figure 1).

Clinical and disease-related variables were systematically retrieved from electronic medical records, including age, sex, Eastern Cooperative Oncology Group (ECOG) performance status, body mass index, comorbidities (including diabetes mellitus), smoking and alcohol use, primary tumor location, metastatic distribution, and first-line systemic treatment history.

### 2.2. Biomarker Definitions and Laboratory Assessment

Baseline laboratory measurements were obtained within 7–10 days prior to the initiation of first-line systemic therapy, thereby minimizing treatment-related variability and ensuring temporal alignment with prognostic assessment.

The following parameters were recorded: absolute neutrophil, lymphocyte, monocyte, and platelet counts (×10^9^/L); C-reactive protein (CRP) (mg/L); albumin (g/dL); and lactate dehydrogenase (LDH) (U/L).

Inflammatory indices were defined as follows: NLR, SII (neutrophils × platelets/lymphocytes), and SIRI (neutrophils × monocytes/lymphocytes). The GINI was calculated according to a previously validated formula: (neutrophils × platelets × monocytes × CRP)/(lymphocytes × albumin) [11,17,18].

These biomarkers were selected to capture the complementary dimensions of tumor–host interaction, encompassing systemic inflammation, immune competence, and nutritional status.

### 2.3. Treatment and Response Assessment

All patients received guideline-concordant systemic therapy in accordance with contemporary international recommendations, including FOLFIRINOX or gemcitabine-based combination regimens. Treatment selection was based on a multidisciplinary evaluation incorporating clinical performance status, comorbidity profile, and disease burden [19].

Tumor response and disease progression were assessed using cross-sectional imaging at regular intervals according to standardized oncologic practices. Radiologic evaluations were performed using contrast-enhanced computed tomography or other clinically indicated modalities and interpreted according to the Response Evaluation Criteria in Solid Tumors (RECIST) version 1.1. This approach ensured consistent and internationally recognized criteria for response assessment across the study population.

Overall survival (OS) was defined as the time from the date of histopathological diagnosis to death from any cause or the last follow-up. Progression-free survival (PFS) was defined as the time from diagnosis to objective disease progression, as determined by the RECIST 1.1 criteria, death, or last follow-up, whichever occurred first.

### 2.4. Statistical Analysis

All statistical analyses were performed using IBM SPSS Statistics (version 27.0; IBM Corp., Armonk, NY, USA) and R software (version 4.5.3; R Foundation for Statistical Computing, Vienna, Austria). Continuous variables are summarized as mean ± standard deviation or median (interquartile range), and categorical variables are summarized as frequencies and percentages. Group comparisons were conducted using the chi-square or Fisher’s exact tests, as appropriate.

Receiver operating characteristic (ROC) curve analysis was used to evaluate the discriminative performance of the inflammatory indices for survival outcomes. The optimal cutoff values were determined using the Youden index. Given the exploratory nature of this approach, cut-offs were derived within the study cohort and consistently applied across analyses to facilitate comparative evaluation. Internal validation of the ROC analysis was performed using 1000 bootstrap resamples to evaluate the robustness and stability of the discriminatory performance estimates.

Survival outcomes were estimated using the Kaplan–Meier method and compared using the log-rank test. Univariate and multivariate Cox proportional hazards regression models were constructed to identify factors associated with OS and PFS. Variables with a *p*-value < 0.10 in the univariable analysis were included in the multivariable models.

Two multivariable models were specified a priori. Model 1 incorporated clinicopathological variables and GINI, whereas Model 2 included NLR to explore the relative contribution of composite versus conventional inflammatory indices. Given the shared biological substrates of these indices, multicollinearity was assessed using variance inflation factors (VIFs), with values > 5 indicating potential collinearity.

The proportional hazards assumption was evaluated using Schoenfeld residuals and graphical methods. Hazard ratios (HRs) with 95% confidence intervals (CIs) were reported. A *p*-values < 0.05 were considered statistically significant.

## 3. Results

### 3.1. Patient Characteristics

A total of 126 patients with metastatic pancreatic cancer were included in the final analyses. The median age distribution was skewed toward an older population, with 57.1% of patients aged ≥65 years, and 55.6% were men. The majority of patients had an ECOG performance status of 0–1 (76.2%), reflecting a predominantly fit population.

Tumors were most frequently located in the head and neck region (64.3%), and liver metastases were present in 74.6% of patients, consistent with the expected metastatic pattern in PDAC. FOLFIRINOX was the most commonly administered first-line regimen (46.0%), followed by gemcitabine-based combinations.

### 3.2. Discriminative Performance of Inflammatory Indices

ROC analysis demonstrated that all evaluated inflammatory indices exhibited statistically significant discriminative abilities for survival outcomes (*p* < 0.05). Among these, GINI exhibited the highest overall performance, with an AUC of 0.769 (95% CI, 0.637–0.900), surpassing NLR, SII, and SIRI.

**Figure 2:**
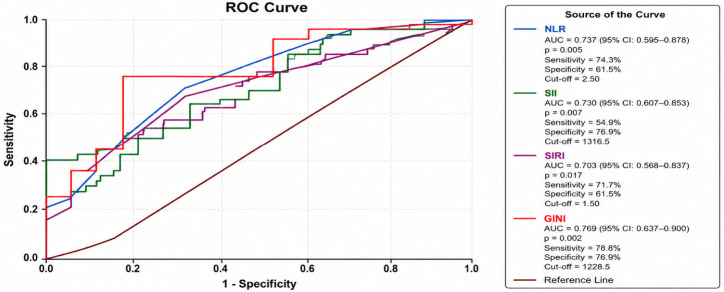
Receiver operating characteristic (ROC) curves of inflammatory indices (NLR, SII, SIRI, and GINI) for predicting clinical outcomes. Abbreviations: AUC, area under the curve; CI, confidence interval; NLR, neutrophil-to-lymphocyte ratio; SII, systemic immune-inflammation index; SIRI, systemic inflammation response index; GINI, Global Immune–nutrition–Inflammation Index.

GINI yielded the highest discriminatory performance among the evaluated inflammatory indices (AUC = 0.769, 95% CI: 0.637–0.900, *p* = 0.002), with an optimal cut-off value of 1228.5. This threshold provided a sensitivity of 78.8% and a specificity of 76.9%. Internal validation using 1000 bootstrap resamples confirmed the stability of the model, with a bootstrap-corrected AUC remaining comparable to the original estimate (Figure 2).

ROC analyses were performed for overall survival at a clinically relevant time horizon.

### 3.3. Association Between GINI and Clinicopathologic Characteristics

Patients were stratified according to their GINI levels using a Youden index-derived cut-off (1228.5). Baseline clinicopathological characteristics were broadly comparable between the low- and high-GINI groups, with no statistically significant differences observed in demographic variables, ECOG performance status, tumor location, or metastatic distribution (*p* > 0.05).

Notably, treatment allocation differed between the groups, with a higher proportion of patients in the low-GINI group receiving FOLFIRINOX (*p* = 0.045). In addition, GINI demonstrated strong associations with other inflammatory indices, as patients with high GINI levels more frequently exhibited elevated NLR, SII, and SIRI values (*p* < 0.001), reflecting shared biological underpinnings (Table 1).

### 3.4. Survival Outcomes According to GINI

At a median follow-up of 18.4 months, 89.7% of the patients died. The median OS for the entire cohort was 8.0 months (95% confidence interval [CI], 6.435–9.565).

**Figure 3:**
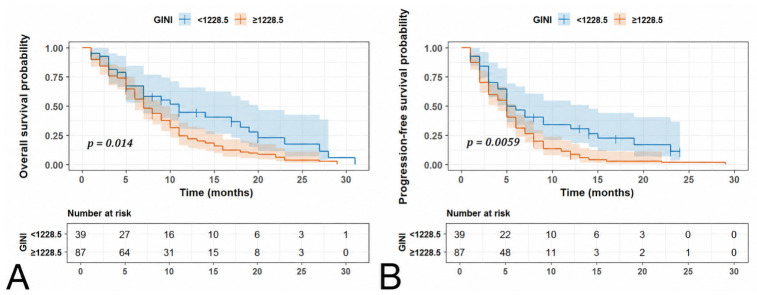
Kaplan–Meier survival curves according to GINI levels. (**A**) Overall survival (OS) and (**B**) Progression-free survival (PFS) stratified by GINI (low vs. ≥high).

Patients with low GINI levels demonstrated significantly improved survival compared to those with high GINI levels. The median OS was 11.0 months (95% CI, 5.960–16.040) in the low-GINI group versus 7.0 months (95% CI, 5.362–8.638) in the high-GINI group (log-rank *p* = 0.014) (Figure 3).

In contrast, although PFS differed significantly between the groups (*p* = 0.006), the median PFS was identical at 5.0 months in both cohorts. This suggests that the observed statistical difference reflects differences in survival distribution rather than a clinically meaningful separation of median outcomes.

### 3.5. Univariable Analysis

In univariable Cox regression analysis for OS, ECOG performance status ≥2 was strongly associated with inferior survival (HR, 3.27; 95% CI, 2.03–5.27; *p* < 0.001). Diabetes was also associated with worse OS (HR, 1.46; 95% CI, 1.01–2.12; *p* = 0.047). Among treatment-related variables, receipt of non-FOLFIRINOX regimens was associated with shorter OS (HR, 1.58; 95% CI, 1.21–2.07; *p* = 0.001). Inflammatory biomarkers demonstrated significant associations with OS. Elevated GINI (≥1228.5) was associated with worse survival (HR, 1.67; 95% CI, 1.08–2.57; *p* = 0.022), as were elevated NLR (≥2.5) (HR, 1.63; 95% CI, 1.04–2.55; *p* = 0.032) and SII (≥1316.5) (HR, 1.53; 95% CI, 1.01–2.33; *p* = 0.045). SIRI was not significantly associated with OS (HR, 1.19; 95% CI, 0.82–1.72; *p* = 0.375). Age, alcohol use, obesity, and metastatic distribution (including liver, lung, and peritoneal involvement) were not significantly associated with OS (*p* > 0.05) (Table 2).

For PFS, ECOG performance status ≥2 was significantly associated with shorter PFS (HR, 1.70; 95% CI, 1.10–2.65; *p* = 0.018). Elevated GINI (HR, 1.75; 95% CI, 1.13–2.71; *p* = 0.012) and NLR (HR, 1.62; 95% CI, 1.04–2.52; *p* = 0.032) were also significantly associated with worse PFS. SII demonstrated a borderline association with PFS (HR, 1.51; 95% CI, 0.99–2.28; *p* = 0.054), whereas the first-line chemotherapy regimen showed a non-significant trend (HR, 1.28; 95% CI, 0.98–1.68; *p* = 0.069). No significant associations were observed between age, diabetes, alcohol use, obesity, metastatic involvement, and SIRI (all *p* > 0.05) (Table 2).

### 3.6. Multivariable Analysis

In the multivariable Cox regression analysis for overall survival, ECOG performance status ≥ 2 remained independently associated with worse survival in both models (Model 1: HR, 2.94; 95% CI, 1.77–4.87; *p* < 0.001; Model 2: HR, 2.85; 95% CI, 1.72–4.74; *p* < 0.001). In Model 1, GINI demonstrated a borderline association with OS (HR, 1.48; 95% CI, 0.94–2.33; *p* = 0.090), whereas the first-line chemotherapy regimen also showed a borderline association (HR, 1.35; 95% CI, 0.99–1.86; *p* = 0.062). No other variables were statistically significant. In Model 2, after the inclusion of NLR, first-line chemotherapy regimen was independently associated with OS (HR, 1.42; 95% CI, 1.03–1.97; *p* = 0.033). Neither GINI (HR, 1.24; 95% CI, 0.74–2.08; *p* = 0.424) nor NLR (HR, 1.43; 95% CI, 0.84–2.41; *p* = 0.186) was independently associated with OS (Table 3).

For progression-free survival, ECOG performance status ≥ 2 remained independently associated with shorter PFS in both models (Model 1: HR, 1.74; 95% CI, 1.08–2.79; *p* = 0.022; Model 2: HR, 1.76; 95% CI, 1.10–2.83; *p* = 0.019). In Model 1, an elevated GINI was independently associated with worse PFS (HR, 1.71; 95% CI, 1.09–2.70; *p* = 0.021). However, in Model 2, this association was attenuated (HR, 1.41; 95% CI, 0.83–2.39; *p* = 0.206), and NLR was not independently associated with PFS (HR, 1.44; 95% CI, 0.84–2.46; *p* = 0.181). No statistically significant associations were observed between age, diabetes, or treatment regimen and PFS (*p* > 0.05) (Table 3).

## 4. Discussion

Metastatic pancreatic cancer remains a paradigmatic example of malignancy in which clinical outcomes are dictated not only by tumor-intrinsic biology but also by the systemic condition of the host [20]. In this context, the identification of biomarkers capable of capturing the multidimensional tumor–host interface is a critical unmet need. In the present study, we demonstrated that the GINI was significantly associated with survival outcomes and exhibited superior discriminative performance compared with conventional inflammatory indices. However, its prognostic contribution appears to be context-dependent and attenuated in multivariable models, highlighting important conceptual and methodological considerations for interpreting composite biomarkers.

The association between elevated GINI and poorer survival appears biologically credible and is consistent with current knowledge of pancreatic cancer biology. GINI reflects several key aspects of the host response, including systemic inflammation (neutrophils, monocytes, and C-reactive protein), immune status (lymphocytes), and nutritional condition (albumin). Together, these parameters point to a state of cancer-related systemic imbalance, marked by enhanced inflammatory activity, weakened antitumor immunity, and metabolic decline [11].

In PDAC, where cachexia, immune suppression, and chronic inflammation often coexist, a composite index such as GINI may provide a more comprehensive picture of disease burden than markers based on a single pathway. This broader representation of the tumor–host interaction likely explains why GINI demonstrated better discriminative performance in our cohort compared with NLR, SII, and SIRI [21,22,23].

Despite favorable ROC and univariable survival results, GINI lost independent prognostic significance after adjustment for key clinical variables, particularly performance status. This finding should not be interpreted as a limitation of the biomarker per se, but rather as an indication of its biological positioning within the prognostic hierarchy. Performance status is a composite clinical construct that encapsulates multiple dimensions of the disease burden, including systemic inflammation, nutritional status, and functional reserve. In this regard, GINI may function as a quantifiable surrogate of the same underlying tumor–host processes that are clinically captured by the performance status. Therefore, the attenuation of its independent effect in multivariable models likely reflects shared biological variance rather than a lack of prognostic relevance. This observation underscores a broader principle in biomarker research: composite indices derived from overlapping physiological domains may enhance risk discrimination while failing to demonstrate statistical independence in conventional regression analyses.

From a methodological perspective, our findings highlight the inherent challenges associated with the evaluation of composite inflammatory biomarkers. Indices such as GINI, NLR, SII, and SIRI are mathematically and biologically interrelated because they are derived from overlapping hematologic parameters [11]. This structural interdependence introduces the potential for multicollinearity and may obscure the independent contribution of individual markers when they are simultaneously included in multivariable models. Although the variance inflation factor analyses in our study did not indicate prohibitive collinearity, the conceptual overlap remains relevant and may partially explain the observed attenuation of effect sizes. In addition, the use of cohort-derived cutoff values, while common in exploratory biomarker studies, introduces the risk of overfitting and may limit generalizability across populations [15]. These considerations reinforce the importance of cautious interpretation and the need for external validation.

An additional important observation in our study is the apparent discrepancy between the statistical significance and clinical relevance of the PFS analyses. Although GINI was associated with PFS in both the univariable and one multivariable model, the absence of a meaningful difference in median PFS between groups suggests that the magnitude of this association may be limited in practical terms. This finding emphasizes the need to distinguish between statistical detectability and clinically actionable effect size, particularly in retrospective datasets with relatively homogeneous outcomes. This further supports the notion that the primary utility of the GINI may lie in risk stratification rather than in predicting treatment-specific benefits.

From a clinical standpoint, the potential value of GINI lies in its ability to refine baseline risk assessment using readily available laboratory parameters. As treatment options for metastatic PDAC continue to expand, such tools may support patient stratification, clinical trial enrichment, and the identification of patients less likely to benefit from intensive treatment [24]. However, given its lack of independent prognostic significance, the GINI should not be viewed as a standalone decision-making tool but rather as a complementary metric that contextualizes established clinical factors.

This study had several limitations that merit consideration. Its retrospective, single-center design introduces the potential for selection bias and limits its external generalizability. The modest sample size may have reduced the statistical power, particularly in multivariable analyses, and may have contributed to the instability of the effect estimates. Inflammatory markers were assessed at a single time point and did not capture dynamic changes during treatment, which may carry additional prognostic information. Furthermore, unmeasured confounders, including subclinical inflammatory conditions and treatment-related factors, could not be excluded. Notably, patients in the low-GINI group were more likely to receive FOLFIRINOX-based chemotherapy, which may partly reflect differences in baseline fitness and treatment selection rather than the independent effect of the biomarker itself. Therefore, the observed survival differences should be interpreted with caution, as treatment-related factors may have contributed to the prognostic separation between groups. Finally, the absence of an independent validation cohort precluded definitive conclusions regarding the reproducibility of our findings.

## 5. Conclusions

GINI is a biologically plausible and clinically accessible composite biomarker that demonstrates superior discriminative performance compared with conventional inflammatory indices in metastatic pancreatic cancer. Although its independent prognostic contribution appears limited, its ability to capture the integrated tumor–host state supports its potential role in risk stratification frameworks. Accordingly, our findings suggest that GINI may be most useful as a tool for baseline risk stratification rather than as an independently validated prognostic marker after adjustment for established clinical factors. Future prospective studies incorporating longitudinal biomarker assessment and external validation are warranted to define its clinical utility and clarify its role within evolving precision oncology paradigms.

## Figures and Tables

**Table 1:** Association between GINI levels and clinicopathologic characteristics.

Variable	Category	Total, *n* (%)	Low GINI (<1228.5), *n* (%)	High GINI (≥1228.5), *n* (%)	*p*
Age	<65	54 (42.9)	16 (41.0)	38 (43.7)	0.468
	≥65	72 (57.1)	23 (59.0)	49 (56.3)	
Sex	Male	70 (55.6)	23 (59.0)	47 (54.0)	0.374
	Female	56 (44.4)	16 (41.0)	40 (46.0)	
Comorbidity	No	40 (31.7)	14 (35.9)	26 (29.9)	0.319
	Yes	86 (68.3)	25 (64.1)	61 (70.1)	
Smoking	No	70 (55.6)	22 (56.4)	48 (55.2)	
	Ex-smoker	22 (17.5)	7 (17.9)	15 (17.2)	0.849
	Yes	34 (27.0)	10 (25.6)	24 (27.6)	
Alcohol use	No	119 (94.4)	38 (97.4)	81 (93.1)	0.302
	Yes	7 (5.6)	1 (2.6)	6 (6.9)	
Obesity	Yes	19 (15.1)	5 (12.8)	14 (16.1)	0.428
	No	107 (84.9)	34 (87.2)	73 (83.9)	
Diabetes	No	71 (56.3)	24 (61.5)	47 (54.0)	0.278
	Yes	55 (43.7)	15 (38.5)	40 (46.0)	
ECOG PS	0–1	96 (76.2)	31 (79.5)	65 (74.7)	0.366
	≥2	30 (23.8)	8 (20.5)	22 (25.3)	
Liver metastasis	No	32 (25.4)	8 (20.5)	24 (27.6)	0.270
	Yes	94 (74.6)	31 (79.5)	63 (72.4)	
Lung metastasis	No	100 (79.4)	32 (82.1)	68 (78.2)	0.404
	Yes	26 (20.6)	7 (17.9)	19 (21.8)	
Peritoneal involvement	No	110 (87.3)	34 (87.2)	76 (87.4)	0.592
	Yes	16 (12.7)	5 (12.8)	11 (12.6)	
Tumor location	Head–neck	81 (64.3)	26 (66.7)	55 (63.2)	0.817
	Tail	27 (21.4)	7 (17.9)	20 (23.0)	
	Body	18 (14.3)	6 (15.4)	12 (13.8)	
First-line chemotherapy	FOLFIRINOX	58 (46.0)	22 (56.4)	36 (41.4)	
	Gemcitabine-based combinations	51 (40.5)	15 (38.5)	36 (41.4)	0.045
	Others	17 (13.5)	2 (5.1)	15 (17.2)	
NLR	<2.5	35 (27.8)	26 (66.7)	9 (10.3)	<0.001
	≥2.5	91 (72.2)	13 (33.3)	78 (89.7)	
SIRI	<1.5	40 (31.7)	28 (71.8)	32 (36.8)	<0.001
	≥1.5	86 (68.3)	11 (28.2)	55 (63.2)	
SII	<1316.5	60 (47.6)	28 (71.8)	12 (13.8)	<0.001
	≥1316.5	66 (52.4)	11 (28.2)	75 (86.2)	

Abbreviations: ECOG PS, Eastern Cooperative Oncology Group performance status; NLR, neutrophil-to-lymphocyte ratio; SII, systemic immune-inflammation index; SIRI, systemic inflammation response index; GINI, Global Immune–Nutrition–Inflammation Index.

**Table 2:** Univariate Cox regression analysis for overall survival and progression-free survival.

Variable	Category	Overall Survival		Progression-Free Survival	
		HR (95% CI)	*p*	HR (95% CI)	*p*
Age	<65 (Ref.)	1	—	1	—
	≥65	1.25 (0.86–1.83)	0.243	1.04 (0.72–1.53)	0.822
ECOG PS	0–1 (Ref.)	1	—	1	—
	≥2	3.27 (2.03–5.27)	<0.001	1.70 (1.10–2.65)	0.018
Diabetes	No (Ref.)	1	—	1	—
	Yes	1.46 (1.01–2.12)	0.047	1.22 (0.84–1.77)	0.307
Alcohol use	No (Ref.)	1	—	1	—
	Yes	1.22 (0.57–2.64)	0.609	1.53 (0.71–3.31)	0.278
Obesity	No (Ref.)	1	—	1	—
	Yes	1.14 (0.65–1.97)	0.652	1.39 (0.80–2.42)	0.240
Peritoneal involvement	No (Ref.)	1	—	1	—
	Yes	1.02 (0.58–1.80)	0.937	1.23 (0.70–2.15)	0.475
Liver metastasis	No (Ref.)	1	—	1	—
	Yes	0.98 (0.64–1.49)	0.922	0.88 (0.58–1.34)	0.551
Lung metastasis	No (Ref.)	1	—	1	—
	Yes	1.26 (0.78–2.04)	0.342	0.82 (0.51–1.32)	0.408
First-line chemotherapy	FOLFIRINOX (Ref.)	1	—	1	—
	Others	1.58 (1.21–2.07)	0.001	1.28 (0.98–1.68)	0.069
NLR	<2.5 (Ref.)	1	—	1	—
	≥2.5	1.63 (1.04–2.55)	0.032	1.62 (1.04–2.52)	0.032
SII	<1316.5 (Ref.)	1	—	1	—
	≥1316.5	1.53 (1.01–2.33)	0.045	1.51 (0.99–2.28)	0.054
SIRI	<1.5 (Ref.)	1	—	1	—
	≥1.5	1.19 (0.82–1.72)	0.375	1.15 (0.79–1.67)	0.470
GINI	<1228.5 (Ref.)	1	—	1	—
	≥1228.5	1.67 (1.08–2.57)	0.022	1.75 (1.13–2.71)	0.012

Abbreviations: HR, hazard ratio; ECOG PS, Eastern Cooperative Oncology Group performance status; NLR, neutrophil-to-lymphocyte ratio; SII, systemic immune-inflammation index; SIRI, systemic inflammation response index; GINI, Global Immune–Nutrition–Inflammation Index.

**Table 3:** Multivariate Cox regression analysis for overall survival and progression-free survival.

Variable	Overall Survival				Progression-Free Survival			
	Model 1 HR (95% CI)	*p*	Model 2 HR (95% CI)	*p*	Model 1 HR (95% CI)	*p*	Model 2 HR (95% CI)	*p*
Age								
<65 (Ref.)	1	—	1	—	1	—	1	—
≥65	0.89 (0.57–1.38)	0.603	0.87 (0.56–1.36)	0.547	0.86 (0.56–1.32)	0.491	0.83 (0.54–1.28)	0.402
ECOG PS								
0–1 (Ref.)	1	—	1	—	1	—	1	—
≥2	2.94 (1.77–4.87)	<0.001	2.85 (1.72–4.74)	<0.001	1.74 (1.08–2.79)	0.022	1.76 (1.10–2.83)	0.019
Diabetes								
No (Ref.)	1	—	1	—	1	—	1	—
Yes	1.22 (0.83–1.80)	0.322	1.18 (0.79–1.74)	0.420	1.07 (0.73–1.57)	0.729	1.01 (0.68–1.50)	0.950
First-line chemotherapy								
FOLFIRINOX (Ref.)	1	—	1	—	1	—	1	—
Others	1.35 (0.99–1.86)	0.062	1.42 (1.03–1.97)	0.033	1.17 (0.86–1.58)	0.319	1.23 (0.90–1.68)	0.193
GINI								
<1228.5 (Ref.)	1	—	1	—	1	—	1	—
≥1228.5	1.48 (0.94–2.33)	0.090	1.24 (0.74–2.08)	0.424	1.71 (1.09–2.70)	0.021	1.41 (0.83–2.39)	0.206
NLR								
<2.5 (Ref.)	—	—	1	—	—	—	1	—
≥2.5	—	—	1.43 (0.84–2.41)	0.186	—	—	1.44 (0.84–2.46)	0.181

Abbreviations: HR, hazard ratio; ECOG PS, Eastern Cooperative Oncology Group performance status; NLR, neutrophil-to-lymphocyte ratio; GINI, Global Immune–Nutrition–Inflammation Index.

## Data Availability

The datasets generated and analyzed during the current study are available from the corresponding author upon reasonable request, subject to approval by the Clinical Oncology Erciyes University Faculty of Medicine.

## Abbreviations

PDAC	Pancreatic ductal adenocarcinoma
GINI	Global Immune–Nutrition–Inflammation Index
NLR	Neutrophil-to-lymphocyte ratio
SII	Systemic immune-inflammation index
SIRI	Systemic inflammation response index
CRP	C-reactive protein
LDH	Lactate dehydrogenase
ECOG PS	Eastern Cooperative Oncology Group performance status
ROC	Receiver operating characteristic
AUC	Area under the curve
OS	Overall survival
PFS	Progression-free survival
HR	Hazard ratio
CI	Confidence interval
SPSS	Statistical Package for the Social Sciences.
RECIST	Response Evaluation Criteria in Solid Tumors
